# Supplementary material for: Effect of Light Intensity and Light Spectrum of LED Light Sources on Photosynthesis and Secondary Metabolite Synthesis in Ocimum basilicum
Source: Plants (Basel). 2025 Apr 28;14(9):1334. doi: 10.3390/plants14091334 (PMC12073442; doi:10.3390/plants14091334)
Supplement: Supplementary file 1 [file plants-14-01334-s001.zip › plants-3570941-supplementary.pdf]

Article

Effect of Light Intensity and Light Spectrum of LED Light Sources on Photosynthesis and Secondary Metabolite Synthesis in *Ocimum basilicum*

Luca Jokic [1,*], Isabell Pappert [1], Tran Quoc Khanh [2] and Ralf Kaldenhoff [1]

[1] Department of Applied Plant Sciences, Faculty of Biology, Technical University Darmstadt, 64287 Darmstadt, Germany; isabell.pappert@tu-darmstadt.de (I.P.); kaldenhoff@bio.tu-darmstadt.de (R.K.)
[2] Department of Adaptive Lighting Systems and Visual Processing, Technical University Darmstadt, 64287 Darmstadt, Germany; khanh@lichttechnik.tu-darmstadt.de
* Correspondence: luca.jokic@tu-darmstadt.de

Supplementary Figures

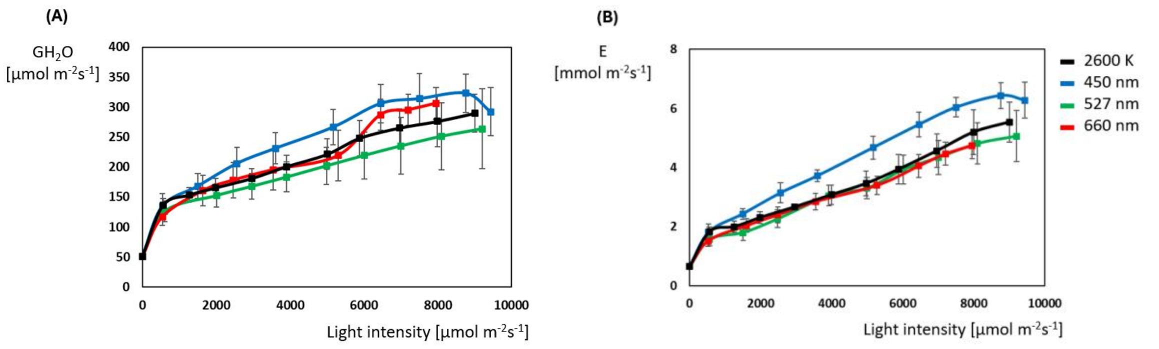

Figure S1. Comparison of (**A**) stomatal conductance (GH_2O) and (**B**) transpiration rate (E) at different illuminations in *Ocimum basilicum* L. (N=10 for each light color).

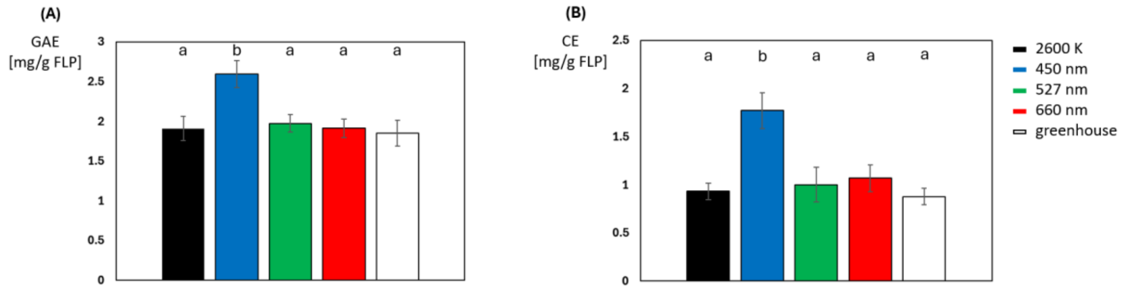

Figure S2. Comparison of (**A**) total phenolic- and (**B**) total flavonoid content expressed in mg gallic equivalents- and mg catechin equivalents per g of frozen leaf powder of greenhouse-grown plants and plants illuminated with increasing light intensities up to 2500 µmol m^{-2} s^{-1} for 90 minutes
